# *In vivo* evidence for translesion synthesis by the replicative DNA polymerase δ

**DOI:** 10.1093/nar/gkw439

**Published:** 2016-05-16

**Authors:** Kouji Hirota, Masataka Tsuda, Mohiuddin, Toshiki Tsurimoto, Isadora S. Cohen, Zvi Livneh, Kaori Kobayashi, Takeo Narita, Kana Nishihara, Junko Murai, Shigenori Iwai, Guillaume Guilbaud, Julian E. Sale, Shunichi Takeda

**Affiliations:** 1 Department of Radiation Genetics, Graduate School of Medicine, Kyoto University, Yoshidakonoe, Sakyo-ku, Kyoto 606-8501, Japan; 2 Department of Chemistry, Graduate School of Science and Engineering, Tokyo Metropolitan University, Minami-Osawa 1-1, Hachioji- shi, Tokyo 192-0397, Japan; 3 Department of Biology, School of Sciences, Kyushu University, 6-10-1 Hakozaki, Higashi-ku, Fukuoka 812-8581, Japan; 4 Weizmann Institute of Science, Department of Biological Chemistry, Rehovot 76100, Israel; 5 Division of Chemistry, Graduate School of Engineering Science, Osaka University, 1–3 Machikaneyama, Toyonaka, Osaka 560-8531, Japan; 6 Medical Research Council Laboratory of Molecular Biology, Francis Crick Avenue, Cambridge CB2 0QH, UK

## Abstract

The intolerance of DNA polymerase δ (Polδ) to incorrect base pairing contributes to its extremely high accuracy during replication, but is believed to inhibit translesion synthesis (TLS). However, chicken DT40 cells lacking the POLD3 subunit of Polδ are deficient in TLS. Previous genetic and biochemical analysis showed that POLD3 may promote lesion bypass by Polδ itself independently of the translesion polymerase Polζ of which POLD3 is also a subunit. To test this hypothesis, we have inactivated Polδ proofreading in *pold3* cells. This significantly restored TLS in *pold3* mutants, enhancing dA incorporation opposite abasic sites. Purified proofreading-deficient human Polδ holoenzyme performs TLS of abasic sites *in vitro* much more efficiently than the wild type enzyme, with over 90% of TLS events resulting in dA incorporation. Furthermore, proofreading deficiency enhances the capability of Polδ to continue DNA synthesis over UV lesions both *in vivo* and *in vitro*. These data support Polδ contributing to TLS *in vivo* and suggest that the mutagenesis resulting from loss of Polδ proofreading activity may in part be explained by enhanced lesion bypass.

## INTRODUCTION

Polδ synthesizes DNA with remarkably high fidelity making only a single error per 10^6^ nucleotides synthesised *in vivo* (1). It achieves this accuracy in two ways. In common with other replicative polymerases, it is able to discriminate very accurately between correct and incorrect base pairs at the polymerase active site (2,3). Second, incorrect nucleotides can be removed by the proofreading nuclease domain of Polδ further increasing overall accuracy by 10- to 60-fold (4). The enzymatic properties of its active site also inhibit Polδ from bypassing many DNA lesions *in vitro* (5). In addition, effective TLS by Polδ will be countered by elimination of nucleotides inserted opposite the damaged base by the proofreading exonuclease activity of the enzyme.

Thus, a prevalent model is that Polδ and Polϵ are unable to bypass DNA lesions, leading to arrest of DNA synthesis with the resulting replication blocks being released by specialized translesion DNA polymerases, such as Polη and Polζ, enzymes that have less spatially constrained active sites and that can thus accommodate the distorted base pairing created by damaged bases (6). While these characteristics allow TLS polymerases to bypass lesions, when coupled with the enzymes’ lack of proofreading activity, their deployment results in a reduction in fidelity of several orders of magnitude compared with Polδ and Polϵ (reviewed in (1)).

Polδ consists of four subunits: POLD1/p125, POLD2/p50, POLD3/p66, and POLD4/p12 (7). Although the Polδ holoenzyme is capable of bypass of some lesions *in vitro* (5,8–11), direct evidence for participation of Polδ in TLS *in vivo* is lacking. The POLD1 subunit contains both the DNA polymerase and 3′ to 5′ proofreading exonuclease domains. Genetic and biochemical studies in budding yeast have indicated that POLD3, a subunit that is not essential for cellular proliferation (12), contributes to TLS as an integral component of Polζ (13–16). POLD3 is also a subunit of both Polδ and Polζ in mammalian cells and it has been proposed that it contributes to TLS through its interaction with Polζ (15–18). However, we recently showed that POLD3 contributes to TLS even in the absence of Polζ. *POLD3*^−/−^ (*pold3*) chicken DT40 cells, but not cells lacking *POLζ*^−/−^ (*polζ*), are deficient in maintenance of replication fork progression along a UV-damaged template, and exhibit an altered pattern of abasic site bypass in the immunoglobulin light chain gene (19). Further, we demonstrated that human POLD3 facilitates abasic site bypass by Polδ *in vitro* by promoting extension from the nucleotide inserted opposite the lesion (19).

We advanced a model suggesting that POLD3 may alter the balance between nucleotide incorporation and proofreading by Polδ, increasing the probability that it could complete TLS. A prediction of this idea is that introducing a proofreading mutation into Polδ should bypass the requirement for the POLD3 subunit and at least partially restore TLS to the *pold3* cell line. In this study we test this hypothesis *in vivo* and *in vitro*. We show that inactivation of the proofreading activity of one allele of POLD1 does indeed restore TLS past UV damage and abasic sites in *pold3* cells but not cells deficient in Polζ. Moreover, expression of proofreading-deficient POLD1 substantially changes the spectrum of mutagenesis arising from TLS past UV damage and abasic sites in POLD3^+^ cells. These observations provide direct evidence that Polδ makes a substantial contribution to TLS *in vivo* and suggests that at least some of the mutagenesis in the absence of the proofreading activity of Polδ, as observed for instance in a subset of cancers, is the result of more proficient lesion bypass by the enzyme.

## MATERIALS AND METHODS

### Cell lines

The generation of *pold3, polζ* and *polη* DT40 single and combination mutants has been described previously (19–22).

### Knock-in of *Pold1^exo−^* mutation

A *pold1^exo-^*mutation knock-in construct was generated from the genomic sequence covering the *POLD1* gene isolated from a genomic library. *POLD1* genomic sequence was isolated from a genomic library by hybridization and a 2.6 kb *Pst*I fragment containing exons10 and 11 cloned into pBlueScript SK. A conserved residue in exonuclease domain, 402Asp (encoded in exon 10) was mutated to Ala using following primers. 5′-CAGAACTTCGCCCTGCCCTAC-3′ and 5′-GTAGGGCAGGGCGAAGTTCTG-3′. This mutation has been previously shown to completely eliminate the exonuclease activity of Polδ *in vitro* (11). The mutation concurrently disrupts a recognition site for a restriction enzyme, *Taq*I. A *HisD* selection-marker gene flanked by loxP sequences was inserted into the *Nde*I site in intron 10 to generate a *pold1^exo-^*mutation knock-in construct. Wild type and *pold3* cells were transfected with *pold1^exo−^HisD*. The 0.1 kb fragment of cDNA covering exon 10 was used as a probe for Southern blot analysis to screen gene-targeting events as previously described (23). The *HisD* selection-marker gene was removed by the transient expression of CRE recombinase. Knock-in of the mutation was confirmed by digestion of the RT-PCR products with *Taq*I. Efficiency of targeting was 31.2% (5/16) and all targeted clones carried the *pold1^exo-^*mutation.

### Sensitivity of cells to genotoxic agents to evaluate DNA repair

Sensitivity of cells to MMS and H_2_O_2_ was measured as a fraction of living cells after proliferation in liquid culture for 48 h. For exposure of cells to MMS or H_2_O_2_, 1 × 10^6^ cells were treated for 1 h at 39.5°C in 1 ml of PBS containing 1% FCS and MMS or complete medium containing H_2_O_2_. 1 × 10^4^ cells were seeded into 24-well plates with 1 ml of medium per well. Plates were incubated at 39.5°C for 48 h. Cell survival was determined using the CellTiter-Glo (Promega). Briefly, 100 μl CellTiter-Glo solution was mixed with 100 μl of cell culture from each well in 96-well plate. After 5 min, luminescence was measured by Fluoroskan Ascent (Thermo).

### AID overexpression by retrovirus infection and analysis of Ig V_λ_ diversification

AID overexpression by retrovirus infection was carried out as described previously (24,25). The efficiency of infection was about 70%, as assayed by GFP expression. Twenty four hours after retrovirus infection, limiting dilution was performed to isolate single colonies. Genomic DNA was extracted at 14 days after limiting dilution from at least three independent colonies. The rearranged V_λ_ segments were PCR amplified using primers 5′-CAGGAGCTCGCGGGGCCGTCACTGATTGCCG-3′, forward in the V_λ_ leader intron, and 5′-GCGCAAGCTTCCCCAGCCTGCCGCCAAGTCCAAG-3′, reverse in the JC_λ_ intron. To minimize PCR-introduced mutation, a high-fidelity polymerase, Prime Star (Takara) was used for amplification. The PCR products were cloned into TOPO Zeroblunt vector (Invitrogen) and sequenced with the M13 forward (−20) primer. Sequence alignment with DNASIS-MAC v3.3 (HITACHI) allowed identification of changes from the consensus sequence of each clone. Mutations were classified as described previously (24,25).

### Dynamic molecular combing and immunofluorescent detection

Asynchronously growing DT40 cells were sequentially labelled for 15 min with 25 μM IdU and for 15 min with 25 μM CIdU. UV treated cells were irradiated at 20 J/m^2^ just before the CldU treatment. At the end of the labelling period (30 min), cells were placed in ice cold 1× PBS (1 volume of cells for 2 volumes of 1× PBS) and centrifuged at 250 g for 5 min at 4°C, washed in ice-cold PBS, and resuspended in PBS to a final concentration of 1 × 10^6^ cells/ml. Three microliters of the cell suspension was spotted onto clean glass Superfrost slides and lysed with 7 μl of 0.5% SDS in 200 mM Tris–HCl (pH 5.5) and 50 mM EDTA (5 min, at room temperature). Slides were tilted at 15° to horizontal, allowing the DNA to run slowly down the slide. Slides were then air dried and fixed in 3:1 methanol/acetic acid, and stored at 4°C before immunolabelling. IdU, CldU, DNA revelations and analysis were performed as described (26), with minor modifications: the DNA was denatured for 30 min in 2.5 N HCl, and CldU was detected using rat anti BrdU (ABD Serotec, Raleigh, NC, USA) at 1/750. A stretching factor of 2.6 for conversion from μm to kb was applied, as previously described for the method in (27). Slides were mounted in 10% 1× PBS and 90% glycerol, kept at −20°C and imaged using a Nikon C1-si confocal microscope.

### PiggyBlock assay

To insert a *Sal*I restriction enzyme site into the original PiggyBlock plasmid (28), we inserted duplex oligonucleotides made of 5′-AATTGGAAGACCCGTCGACCA-3′ and 5′-TATGGTCGACGGGTCTTCC-3′ into the *Mfe*I/*Nde*I sites (piggyBlock-*Sal*I). A 30-nucleotide oligonucleotide, CTCGTCAGCATC(TT)CATCATACAGTCAGTG carrying CPD on (TT), and a 16-nucleotide oligonucleotide, TCGAGCGACACTGGAT, was annealed with complementary 46-nucleotide oligonucleotide, AATTCACTGACTGTATGATGGCGATGCTGACGAGATCCAGTGTCGC. To make piggyBlock-op plasmid, CTCGTCAGCATC(TT)CATCATACAGTCAGTG and TCGAGCGACACTGGAT were annealed with AATTCACTGACTGTATGATG(TT)GATGCTGACGAGATCCAGTGTCGC. The resultant duplex fragment carrying a single CPD lesion was ligated with the piggyBlock-*Sal*I plasmid digested with *Mfe*I/*Sal*I, and ligated plasmid was gel purified (Qiagen), as previously described (28). Ten ng of the ligated plasmid together with 1 μg of the transposase expression vector was transfected into DT40 cells using the Neon transfection system (Invitrogen, CA, USA) with settings, 1350 V, 10 m sec, and three pulses. Transfected cells were subjected to limiting dilution immediately after transfection. Puromycin was added at 30 h after transfection. Genomic DNAs from individual puromycin resistant clones were purified, and were PCR amplified using primers (ACTGATTTTGAACTATAACGACCGCGTGAG) and (ACTAGTGAGACGTGCTACTTCCATTTGTCA) to examine DNA sequences at the CPD lesion. If a single puromycin resistant clone contained two different sequences, we counted as two independent DNA synthesis events. We obtained sequences from *xpa, polη/polζ/xpa* and *polη/polζ/xpa/pold1^exo−^* cells, respectively. We analyzed them following the method described previously (28).

### Protein purification and primer extension assays

The human Polδ holoenzyme, with N-terminal His-tagged p50, was expressed using a baculovirus vector (pBacPAK9, Clontech, Palo Alto, CA, USA) in insect cells (High Five, Life Technologies, Palo Alto, CA), as described previously (29). To inactivate proofreading exonuclease activity, p125 Asp 402 was replaced by Ala. For primer-extension analysis, DNA synthesis was carried out with 0.06 pmol ^32^P-labeled primer. For examining abasic site bypass, 17 mer primer (AGCTATGACCATGATTA) annealed with a 49-mer template oligo DNA (AGCTACCATGCCTGCACGAATXAAGCAATTCGTAATCATGGTCATAGCT), where X can be an abasic site were used. For examining CPD baypass, 16 mer primer (CACTGACTGTATGATG) annealed with 30 mer oligo DNA (CTCGTCAGCATC(TT)CATCATACAGTCAGTG), where (TT) can be CPD were used as illustrated in (11). The assay was carried out in a reaction mixture (5 μl) containing 30 mM HEPES–NaOH (pH 7.4), 7 mM MgCl_2_, 8 mM NaCl, 0.5 mM dithiothreitol, and 10 μM each dNTP in the presence of 15 nM of primer/template complex, 2 nM of Polδ and 50 nM of PCNA for 15 min at 37°C. At the end of the reaction, the products were denatured with formamide and loaded onto 15.6% polyacrylamide gels containing 7 M urea in TBE buffer (89 mM Tris, 89 mM boric acid, 2 mM EDTA). After electrophoresis, radioactivity was measured with a Fuji Image analyzer, FLA2500 (Fujifilm, Tokyo, Japan).

## RESULTS

### Expression of proofreading-deficient Polδ rescues the DNA damage hypersensitivity of pold3 cells

**Figure 1.**
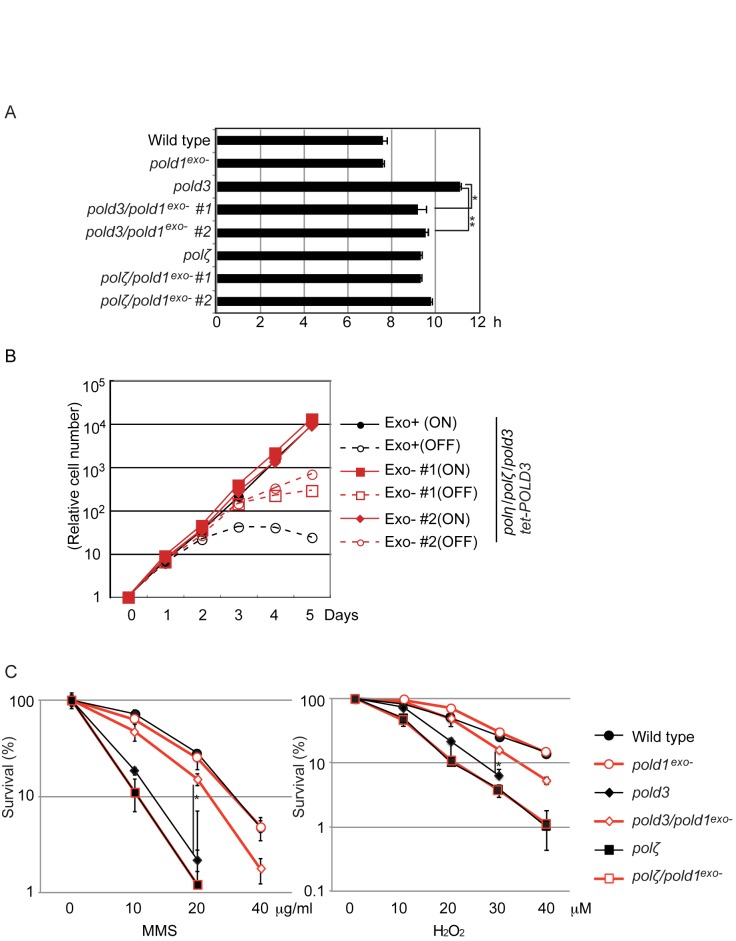
*pold1*^*exo-*^ mutation restores mutant phenotype of *pold3* cells. (**A**) *pold1^exo-^* mutation significantly suppresses the growth defect of *pold3* cells. The doubling time for the indicated genotypes is indicated. Error bars represent standard deviations (SD) from three independent assays. Statistical significance was determined by a Student's *t*-test and *P*-value was calculated. (*) *P* < 0.01, (**) *P* < 0.001. (**B**) Expression of *POLD1^exo−^* reverses the synthetic lethality of *polη/polζ/pold3* cells. Growth curves of the indicated cells are shown after addition of doxycycline at time zero. The *tet-POLD3* transcription was active without doxycyclin (ON) and inhibited upon addition of doxycyclin (OFF). (**C**) Expression of *POLD1^exo−^* reverses sensitivities of *pold3* cells to MMS and H_2_O_2_. Indicated cells were exposed to MMS or H_2_O_2_. The dose of the genotoxic agent is displayed on the x-axis on a linear scale, while the percentage fraction of surviving cells is displayed on the y-axis on a logarithmic scale. Error bars show the SD for three independent assays. Statistical significance was determined by a Student's *t*-test and *P*-value was calculated. (*) *P* < 0.01.

To test the hypothesis that expression of proofreading-defective Polδ would suppress the TLS defect of *pold3* cells, we inserted a point mutation (D402A) into one of the two allelic *POLD1* loci in wild type cells as well as in *pold3* and *polζ* mutant DT40 cells. This generated *pold1*^exo−^, *pold3/pold1^exo−^* and *polζ/pold1^exo−^* cells (Supplementary Figure S1A–D). As expected, *pold1*^exo−^ cells were viable and grew normally (Figure 1A). Interestingly, expression of proofreading deficient Polδ partially normalized the slow proliferation of *pold3* cells, but not that of *polζ* cells (Figure 1A).

We have previously shown that cells lacking both POLD3 and two major TLS polymerases, Polη and Polζ, are inviable (19). We inserted the *pold1*^exo−^ mutation into *polη/polζ/pold3* cells, in which viability was supported by expression of a *POLD3* transgene under the control of the doxycycline-repressible promoter (*tet-POLD3* transgene). The resulting *polη/polζ/pold3/tet-POLD3* cells stopped proliferating on the third day after addition of doxycycline, as previously reported (19) (Figure 1B). However, the *pold1*^exo−^ mutation significantly improved the viability of the triple mutant, *polη/polζ/pold3* cells after addition of doxycycline, but not *polη/polζ* cells (Figure 1B).

We next asked whether expression of proofreading-deficient Polδ enhanced the tolerance of *pold3* and *polζ* cells to DNA damaging agents. *pold3/pold1^exo−^* cells displayed significantly increased tolerance to the alkylating agent methyl methane sulfonate (MMS) and the oxidising agent H_2_O_2_, compared with *pold3* cells (Figure 1C). In contrast, *polζ* and *polζ/pold1^exo−^* cells exhibited indistinguishable sensitivity to MMS and H_2_O_2_. Thus, Polδ proofreading significantly contributes to the DNA damage sensitivity of the *pold3* mutant cells. The reversion of the mutant phenotype associated with *pold3* but not *polζ* by the *pold1^exo−^* mutation suggests that the proofreading activity counteracts TLS by Polδ but not TLS by Polζ.

### Expression of proofreading-deficient Polδ affects TLS past abasic sites in Ig V gene

To test the role of Polδ in TLS *in vivo*, we examined the diversification of the immunoglobulin (Ig) Vλ region in the DT40 B cell line during *in vitro* passage. DT40 cells constitutively diversify their Ig VJλ segment through two mechanisms, TLS dependent hypermutation and gene conversion from upstream pseudo-Vλ segments (30,31). Hypermutation at C/G basepairs in this locus is caused by TLS across abasic sites (32,33). Thus, the nucleotide sequence analysis of Ig V diversification during clonal expansion of cells provides the opportunity for measuring the rate of TLS as well as identifying the nucleotides inserted opposite to abasic sites (20).

**Figure 2.**
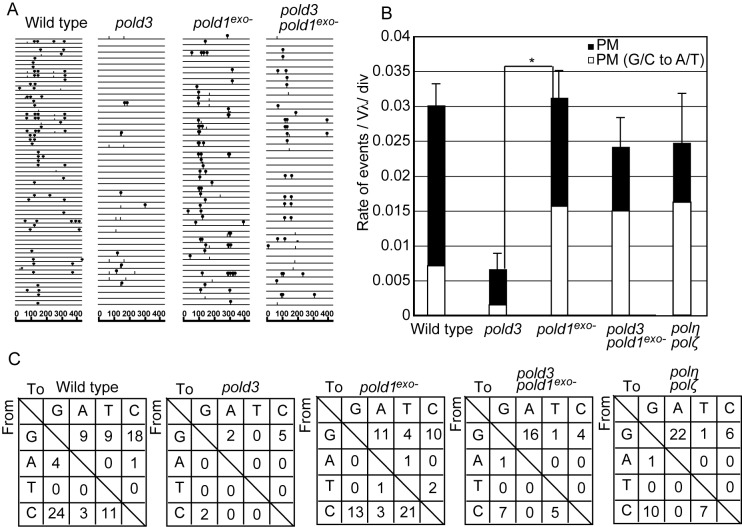
Expression of the proof reading exonuclease deficient Polδ substantially changes the mutation spectrum of the Ig V_λ_ hypermutation. (**A**) Ig V_λ_ segments isolated from indicated cells, clonally expanded for two weeks. Horizontal lines represent the rearranged Ig V_λ_ (450 bp), with hypermutation (lollipop shapes), single-nucleotide substitutions that could be the result of hypermutation or gene conversion (vertical bars). At least three cellular clones were expanded for two weeks and analyzed for each data set. (**B**) The rates of TLS-dependent hypermutation (PM) are indicated with standard error. White bars represent the rate of G/C to A/T mutations, TLS following A-rule. Data from *polη/polζ* cells are taken from (19) for comparison. Statistical significance was determined by a Fisher**’**s exact test and *P*-value was calculated. (*) *P* < 0.05. (**C**) Pattern of point mutation in wild type, *pold3, pold1^exo^*^−^ and *pold3/pold1^exo-^* cells. Tables showing the pattern of mutation in each line. Some of data for wild type, *pold3* and *polη/polζ* cells are from (19).

We overexpressed AID to enhance Ig V diversification. The resultant AID overexpressing cells were subcloned and cultured for two weeks. Then we subjected PCR-amplified VJλ segment to nucleotide sequence analysis (Figure 2A). *pold3* cells exhibited a significant decrease in the rate of TLS dependent hypermutation as reported previously (19) (Figure 2B). Remarkably, TLS dependent hypermutation was restored in *pold3/pold1^exo−^* cells to a nearly wild-type level (Figure 2B). Thus, loss of proofreading exonuclease activity of Polδ bypasses requirement of POLD3 to execute TLS past abasic site (15,16). Interestingly, the restoration was associated with an increase in the proportion of G/C to A/T transitions (Chi-square test, *P* = 0.0050, Figure 2B and C). Polδ preferentially incorporates dA opposite abasic sites (A-rule (34)) while Rev1 preferentially incorporates dC (C-rule (35)). Importantly, the presence of a proofreading deficient allele of Polδ also increases the proportion of dA incorporation opposite C even when POLD3 is present (Chi-square test, *P* = 0.0068, Figure 2C and C). The reversal of the abasic site TLS defect of the *pold3* mutant and marked bias towards G/C to A/T transitions (Figure 2B and C) induced by expression of proofreading-deficient Polδ supports the idea that Polδ can perform TLS past abasic sites *in vivo* and that this is facilitated by the POLD3 subunit.

### Expression of proofreading-deficient Polδ rescues the attenuated replication fork progression of pold3 cells after UV irradiation

**Figure 3.**
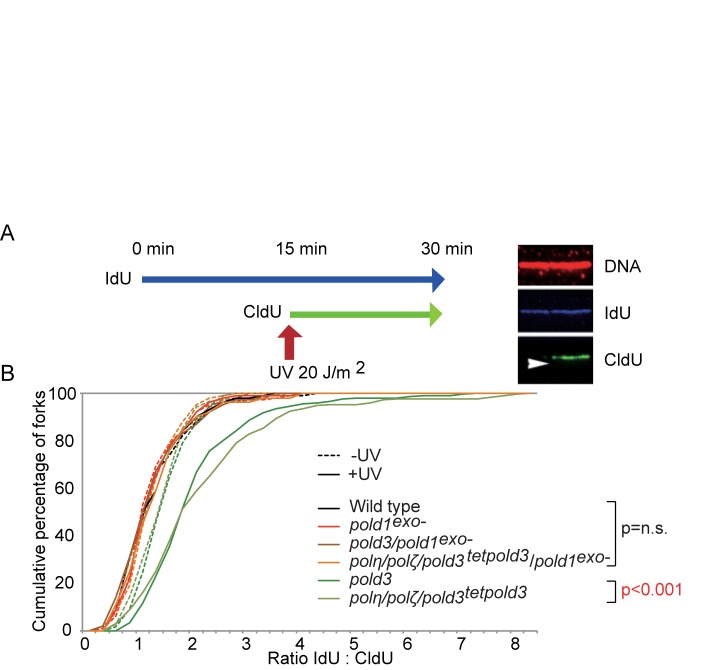
Expression of *POLD1^exo−^* restores defective replication fork progression past UV damaged DNA in *pold3* cells. (**A**) Schematic for DNA fiber labelling. DT40 cells were labeled sequentially with IdU and CldU with or without UV treatment after IdU labeling. The right hand panel shows an example of an ongoing fork. The arrowhead indicates the direction of replication. (**B**) The data for cells carrying the indicated genotypes was plotted as a cumulative percentage (y-axis) of forks at each ratio (x-axis). The transcription of *tet-POLD3* was repressed by doxycycline for 1 day. The *P*-values of the Kolmogorov-Smirnov test for ratio distribution of each mutant for UV compared to wild type are indicated. n.s.: not significant. A part of data for *pold3* and *polη/polζ/pold3* cells were from (19).

Previous work in DT40 cells has revealed temporally separated modes of lesion bypass. One mechanism is responsible for timely filling of postreplicative gaps at UV-damage sites, while another operates at or very close to stalled replication forks and maintains normal fork progression on UV-damaged DNA (36). *pold3* cells are deficient only in the latter mode of damage bypass (19), which can be assessed by DNA molecular combing (Figure 3A). To examine replication fork progression after UV irradiation, we labeled nascent strands with IdU for 15 min, irradiated the cells with UV, and then continued labeling the nascent strands with CldU for a further 15 min (Figure 3A). After DNA combing, we detected the tracts of CldU and IdU with immunofluorescence and calculated the ratio between them to compare the total DNA synthesized before and after UV exposure on a fork-by-fork basis. Counterstaining the fibers for DNA allowed us to distinguish fork stalls from broken DNA. We plotted the data as a cumulative percentage of forks at each ratio (Figure 3B).

*pold3* and *polη/polζ/pold3* cells exhibited a significant reduction in the DNA synthesized during labeling period after UV, as reported previously (19). Interestingly, inactivation of Polδ proofreading significantly restored the defective fork progression of *pold3* cells after UV (Figure 3B), suggesting that the defective TLS of *pold3* cells is suppressed by proofreading-deficiency of the Polδ catalytic subunit. Likewise, the inactivation of Polδ proofreading activity completely restored the UV-induced fork progression defect of *polη/polζ/pold3* cells (Figure 3B), indicating that POLD3 is operating independently of Polη or Polζ in this context. We therefore conclude that Polδ proofreading-deficiency alleviates the *in vivo* TLS defect induced by loss of POLD3, which in turn does not depend on Polη or Polζ.

### Expression of proofreading-deficient Polδ alters pattern of UV induced mutagenesis

**Figure 4.**
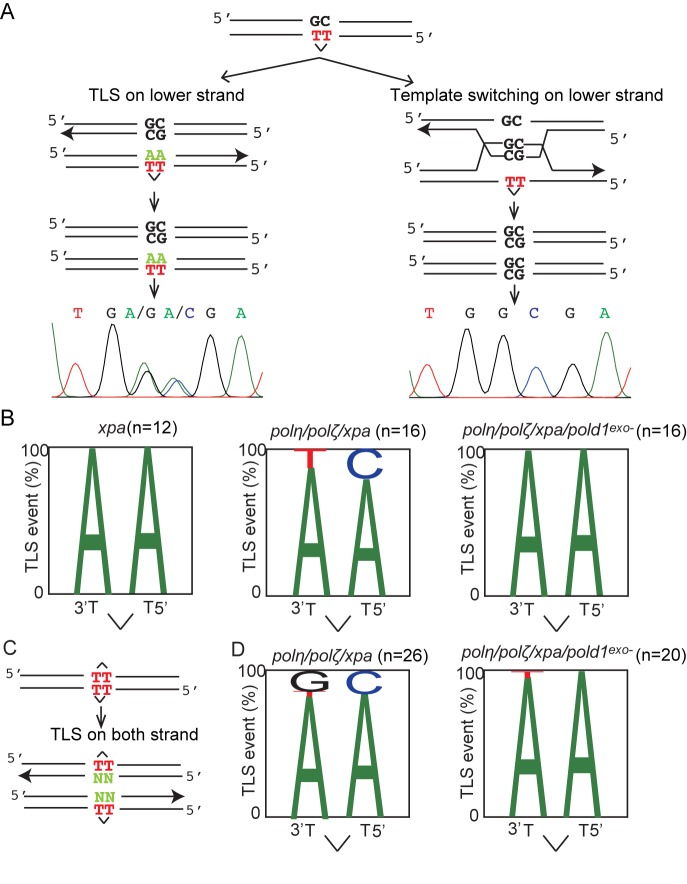
Expression of *POLD1^exo−^* changes the mutation spectrum of TLS past CPD. (**A**) A CPD placed opposite GpC mismatch was randomly integrated into the genome using the PiggyBlock vector. TLS across the CPD results in a dual peak in the resulting cellular clone (left), while template switching results in a homogenous GC read (right). (**B**) The pattern of nucleotide incorporation opposite the CPD site. The percentage of each nucleotide incorporated at each position is indicated by the size of the letter of the nucleotide in the column. The total numbers of TLS events are shown. (**C**) A schematic representation of the opposed arrangement of CPD photoproducts in the piggyBlock plasmid (piggyBlock-op) and possible outcomes of DNA replication, only by TLS, over the lesion. (**D**) The pattern of nucleotide incorporation opposite the CPD in *polη/polζ/xpa* and *polη/polζ/xpa/pold1^exo−^* cells in piggyBlock-op plasmid.

We next investigated whether expression of proofreading-deficient Polδ alters the pattern of TLS-induced mutagenesis at a UV damage (cyclobutane pyrimidine dimer (CPD)) integrated into chromosomal DNA. To this end, we inserted a CPD into the ‘piggyBlock’ transposon-based vector (28), transfected the CPD-carrying vector into the cells and picked individual clones having randomly integrated it (Figure 4A and Supplementary Figure S2). To avoid elimination of the integrated CPD by nucleotide excision repair and TLS by Polη and Polζ, we performed all experiments in *polη/polζ/xpa* background. We analyzed individual clones. These clones were mosaics as the cells within the clone inherit either the Watson or Crick strand of the parental integrant (Figure 4A and Supplementary Figure S2). Thus, in this assay, release of replication block at the CPD site by error-free template switching and by TLS can be distinguished as the CPD-containing TpT is placed opposite a GpC. Template switching would result in GpC at the CPD site, while TLS would insert ApA (accurate TLS) or other bases (inaccurate TLS) at the site (note, insertion of GpC opposite the T-T CPD would be unusual. (13,37)). Accordingly, TLS events are expected to give rise to a dual peak in the sequencing fluorogram (Figure 4A). Template switching, on the other hand, proceeds through the base opposite the lesion, and consequently, its signature consists of a single peak.

Based on this principle, we determined the frequency of TLS relative to template switching (Supplementary Figure S3). We observed a decrease in the use of TLS from 35% to 5.8% following disruption of both *POLη* and *POLζ* in *xpa* cells. Further, the proportion of accurate TLS (i.e. incorporation of ApA) decreased from 100% in *xpa* cells to 85% in *polη/polζ/xpa* cells (Figure 4B). Thus, as expected, Polη and Polζ contribute significantly to accurate TLS past CPDs. Importantly, the expression of proofreading-deficient Polδ in *polη/polζ/xpa* increased the proportion of accurate TLS from 85% to 100% (Figure 4B). Thus, proofreading-deficient Polδ may contribute to the residual TLS past this UV lesion in cells lacking Polη and Polζ. To confirm this conclusion, we designed another piggyBlock vector, piggyBlock-op, in which the lesions are placed non-physiologically opposite each other, a configuration that forces bypass to be executed only by TLS (Figure 4C). Using this approach, we also observed an increase in the proportion of accurate TLS in *polη/polζ/xpa/pold1^exo−^* cells in comparison with *polη/polζ/xpa* cells (Figure 4D). These data support the idea that Polη and Polζ are the primary enzymes responsible for TLS past CPDs, but that Polδ can also contribute to bypass of this lesion, particularly in the absence of these canonical TLS enzymes.

### Proofreading-deficiency causes a dramatic increase in the efficiency of TLS by purified human Polδ holoenzyme

**Figure 5.**
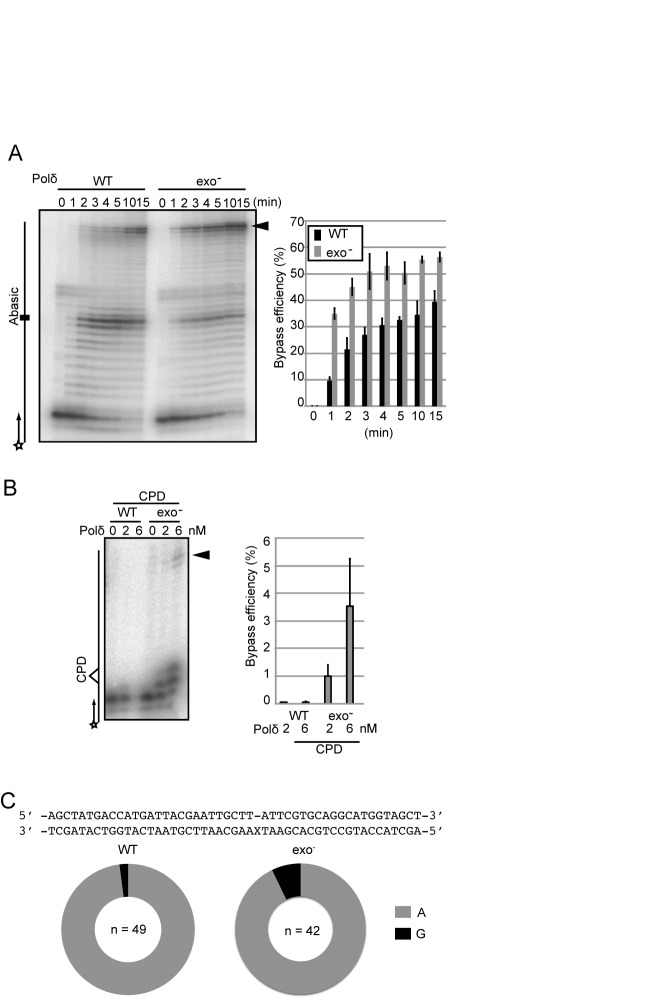
Inactivation of proofreading activity significantly increases the efficiency of Polδ (POLD3^+^) to perform TLS past abasic sites and UV damage. (**A**) DNA synthesis reactions were carried out with 2 nM of proofreading proficient (WT) or deficient (exo^−^) Polδ holoenzyme for the indicated duration. The histogram shows amounts of the fully extended product at the indicated time points. Error bars show the SD for three independent assays. (**B**) DNA synthesis reactions carried out with the indicated Polδ holoenzymes on template and primer strands, which are schematically shown on the left. Amount of the fully extended product was analyzed at 15 min. Error bars show the SD for three independent assays. (**C**) The pie charts indicate percentage of the nucleotides inserted opposite abasic site.

We next tested the capability of purified proofreading-deficient and proofreading-proficient Polδ to perform TLS. Using a physiological concentration (10 μM) of deoxynucleotides, both proofreading-deficient and proofreading-proficient Polδ exhibited comparable efficiency of DNA synthesis over intact template strands. We then analyzed TLS using three sets of primer and template strands, two containing an abasic site and the other one containing CPD (Supplementary Figure S4). We optimized the concentration of Polδ for the *in vitro* DNA synthesis analysis, and decided to use 2 and 6 nM (Supplementary Figure S5A). The efficiency of TLS was evaluated by measuring the amount of fully synthesized products as a function of time (Figure 5A, B; arrowhead). Proofreading-proficient Polδ generated more prominent bands corresponding to stalling at the abasic site, and one nucleotide before site than did the proofreading-deficient enzyme (Figure 5A and Supplementary Figure S5B), suggesting that proofreading-proficient Polδ repeats futile cycles of incorporation and proofreading. Consistent with our *in vivo* observations, proofreading-deficient Polδ performed TLS past an abasic site with a few times higher efficiency than proofreading-proficient Polδ (Figure 5A). To confirm which nucleotides are inserted opposite the abasic site, we purified the DNA synthesis products using a biotinylated primer, PCR amplified them and determined the nucleotide sequence. We did not detect any DNA slippage events in either case. The percentage of DNA synthesis products following the A-rule was 98% and 93% for proofreading-proficient and deficient Polδ, respectively (Figure 5C). This is consistent with the increase in the proportion of the A-rule mutations observed in *pold1^exo−^* cells in comparison with wild type cells (Figure 2B and C). Lastly, we measured TLS past a CPD site. We found that proofreading-deficient Polδ performed TLS with higher efficiency than proofreading-proficient Polδ (Figure 5B). These observations indicate that proofreading-deficiency significantly enhances the capacity for Polδ to perform TLS past abasic sites and CPDs *in vitro*.

## DISCUSSION

Testing the role of Polδ in TLS directly by inactivating the catalytic activity of the enzyme is not possible due to the essential role of Polδ in DNA replication. Several studies have shown that Polδ is capable of lesion bypass *in vitro* (5,8,10,11,38). However, it has remained unclear whether this replicative polymerase contributes significantly to translesion synthesis *in vivo*. Here we provide a number of lines of evidence that provide the strongest evidence to date that it does. First, the POLD1 proofreading deficiency is able to suppress the defect in translesion synthesis caused by loss of POLD3, but not Polζ (Figure 1C), which plays a critical role in completing TLS by extending DNA synthesis after other TLS polymerases have inserted nucleotides opposite damaged template bases (20,39). Thus, the data shown in Figure 1C indicate that when completion of TLS is inefficient due to the loss of Polζ, the POLD1 proofreading activity cannot substitute and rescue the damage sensitivity of *polζ* cells. While the proofreading activity of Polδ has been shown to be able to operate in *tran*s for replication errors (40,41), these observations suggest that POLD1 proofreading acts preferentially on TLS nucleotide incorporation events mediated by POLD1 rather than by other TLS polymerases. Consistent with this, inactivation of POLD1 proofreading activity *in vitro* increases the efficiency of lesion bypass by the Polδ holoenzyme (Figure 5). Further, analysis of the pattern of abasic site bypass in the immunoglobulin light chain locus shows that inactivation of POLD1 proofreading significantly increases the frequency of dA incorporation consistent with POLD1 proofreading its own translesion synthesis nucleotide incorporations.

Given the ability of Polδ to perform error-prone TLS, an interesting question is whether Polδ can modulate its fidelity and proofreading activity when it encounters template damage. We have previously argued that POLD3 may facilitate the ability of Polδ to complete TLS by allowing extension from a base incorporated opposite a lesion (19). We suggested that in the absence of POLD3, Polδ may undergo futile cycles of incorporation and proofreading resulting in its stalling at a lesion. The observations we present here are consistent with this model, by showing that loss of proofreading facilitates completion of TLS by Polδ *in vivo* and *in vitro*. This ability of Polδ to alter its catalytic properties so as to carry out TLS, and therefore to act mutagenically, is at odds with its role as a replicative polymerase. It suggests that the catalytic site and proofreading need to be regulated when the enzyme encounters a lesion. How this ‘fidelity switch’ is regulated remains to be explored, but might be promoted by post-translational modification, for instance of POLD3 itself (42,43). Alternatively, given the proposed role of POLD3 as an anchor between POLD1 and PCNA (44), it may be due to a constrained ‘TLS mode’ interaction of the enzyme with the clamp. Another intriguing possibility is that the TLS ability of the replicative polymerases is regulated local modulation of dNTP concentration. TLS by replicative polymerases in eukaryotic cells has been invoked to explain the increased mutation frequency following exposure to 4-nitro-quinoline oxide (4-NQO) of *S. cerevisiae* in which all TLS polymerases had been deleted (45). DNA damage increases dNTP concentration and this increase promotes lesion bypass by Polϵ, which at normal physiological dNTP concentrations is unable to carry translesion synthesis (45). Since the proofreading exonuclease activity of replicative polymerases is also suppressed by elevated concentration of dNTP (19), it is possible that locally elevated dNTP concentrations in the vicinity of DNA damage allows the replicative polymerases to engage in damage bypass. Supporting of this view, inactivation of exonuclease activity of *E.coli* PolIII (Polδ homolog) enhances bypass replication (46,47).

Our data suggest that bypass by Polδ may be a relatively frequent event, at least at abasic sites and CPDs. Important questions for future studies concern the order in which Polδ and the canonical TLS polymerases are deployed in lesion bypass, and whether Polδ can contribute to TLS equally at leading and lagging strand obstacles. We previously showed that A-rule mutagenesis is also increased in *polη/polζ* cells in comparison with wild type cells, suggesting that Polδ serves as a backup for Polη–Polζ axis (20). Thus, if TLS polymerases fail to restore DNA replication, Polδ might attempt TLS as a last resort. Alternatively, since Polδ is likely to be the first enzyme to encounter a template strand lesion, TLS by Polδ opposite a weakly blocking lesion may well be the most pragmatic mechanism to ensure maintenance of processive replication. In this model, whether the classical TLS apparatus is deployed may depend on whether Polδ can complete the reaction in a reasonable time, or on other contextual cues surrounding the lesion (48). Thus, it will be interesting to determine whether Polδ is used for TLS on both leading and lagging strands, given its prominent role as the lagging strand replicase (49) and a possible interplay among yeast replicative DNA polymerases δ and ϵ (50).

In summary, to completely replicate the whole genomic DNA in a timely fashion, cells have evolved multiple mechanisms, including the firing of dormant replication origins, homologous recombination, and TLS polymerases. Our study provides an insight into a fourth mechanism, bypassing lesions directly with replicative DNA polymerases.

## Supplementary Material

SUPPLEMENTARY DATA
