# Supplementary material for: In vivo evidence for translesion synthesis by the replicative DNA polymerase δ
Source: Nucleic Acids Res. 2016 May 16;44(15):7242–50. doi: 10.1093/nar/gkw439 (PMC5009730; doi:10.1093/nar/gkw439)
Supplement: SUPPLEMENTARY DATA [file supp_44_15_7242__index.html]

In vivo evidence for translesion synthesis by the replicative DNA polymerase δ — In vivo evidence for translesion synthesis by the replicative DNA polymerase δ — In vivo evidence for translesion synthesis by the replicative DNA polymerase δ — SUPPLEMENTARY DATA 

# *In vivo* evidence for translesion synthesis by the replicative DNA polymerase δ

## SUPPLEMENTARY DATA

- SUPPLEMENTARY DATA
