# Supplementary material for: In vivo evidence for translesion synthesis by the replicative DNA polymerase δ
Source: Nucleic Acids Res. 2016 May 16;44(15):7242–50. doi: 10.1093/nar/gkw439 (PMC5009730; doi:10.1093/nar/gkw439)
Supplement: SUPPLEMENTARY DATA [file supp_gkw439_nar-00667-h-2016-File007.pdf]

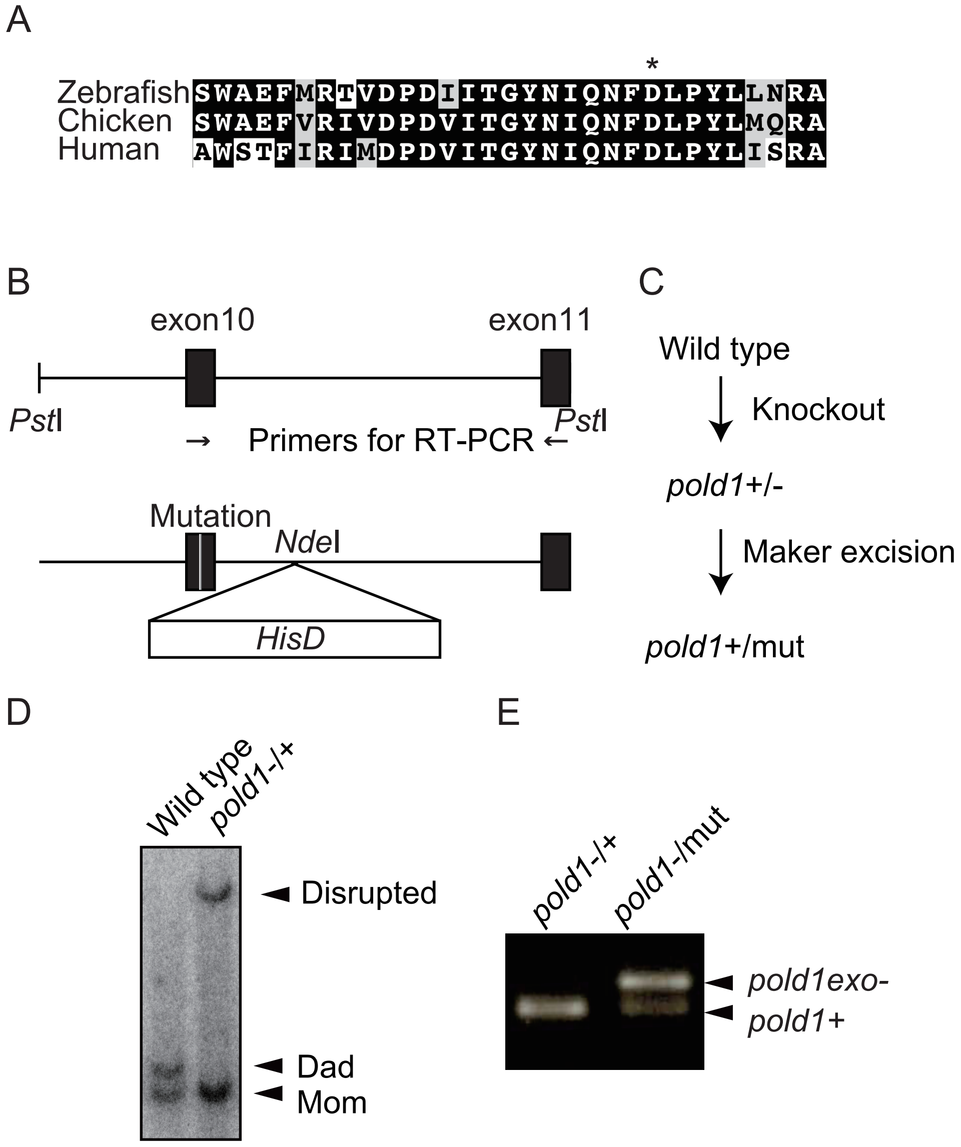
A
*
Zebrafish SWAEFMRTVDPDIITGYNIQNFDLPYLLNRA
Chicken SWAEFVRIVDPDVITGYNIQNFDLPYLMQRA
Human AWSTFIRIMDPDVITGYNIQNFDLPYLISRA
B
exon10
exon11
PstI
PstI
→ Primers for RT-PCR ←
Mutation
NdeI
HisD
C
Wild type
Knockout
pold1+/-
Maker excision
pold1+/mut
D
Wild type
pold1-/+
Disrupted
Dad
Mom
E
pold1-/+
pold1-/mut
pold1exo-
pold1+

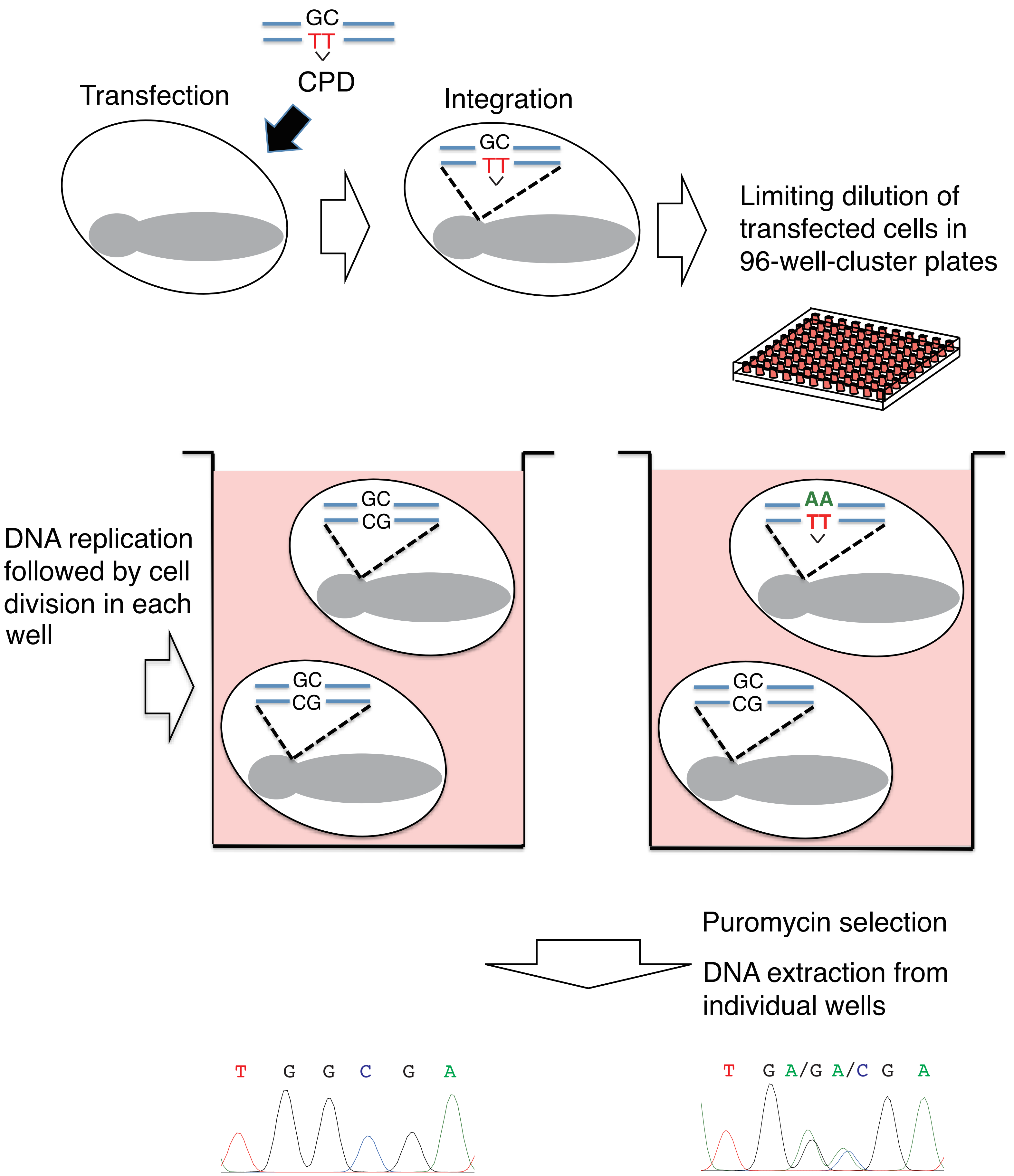
GC
TT
CPD
Transfection
Integration
GC
TT
Limiting dilution of transfected cells in 96-well-cluster plates
DNA replication followed by cell division in each well
GC
CG
GC
CG
AA
TT
GC
CG
Puromycin selection
DNA extraction from individual wells
T G G C G A
T G A/G A/C G A

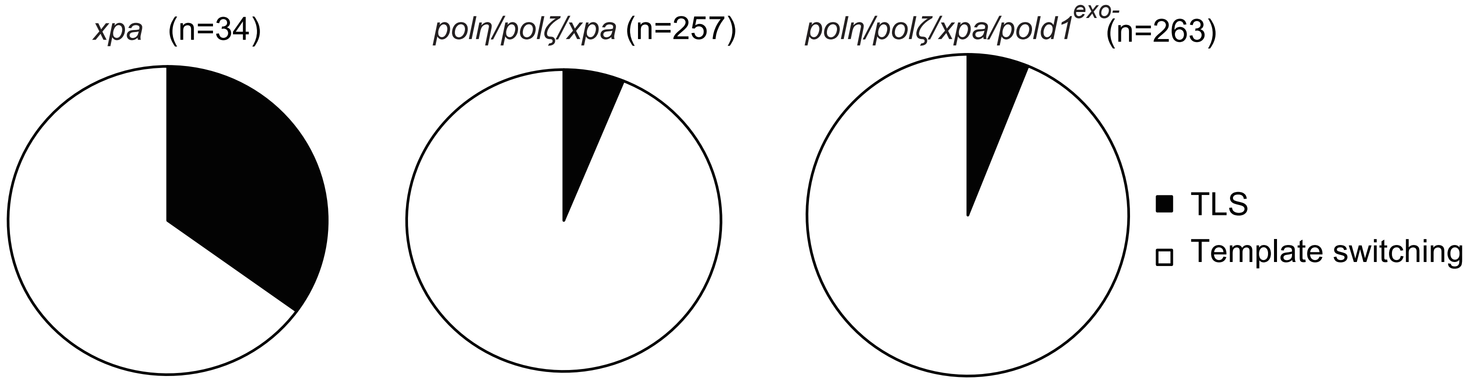
xpa (n=34)
polη/polζ/xpa (n=257)
polη/polζ/xpa/pold1^{exo-} (n=263)
TLS
Template switching

A Abasic template/primer

```
*5' -AGCTATGACCATGATTA-3'
 3' -TCGATACTGGTACTAATGCTTAACGAA(AP)TAAGCACGTCCGTACCATCGA-5'
```

B Abasic template/primer (no slippage)

```
*5' -AGCTATGACCATGATTA-3'
 3' -TCGATACTGGTACTAATGCTTAACGAA(AP)CAAGCACGCCCGCACCACCGA-5'
```

C CPD template/primer

```
*5' -CACTGACTGTATGATG-3'
 3' -GTGACTGACATACTACTTCTACGACTGCTC-5'
```

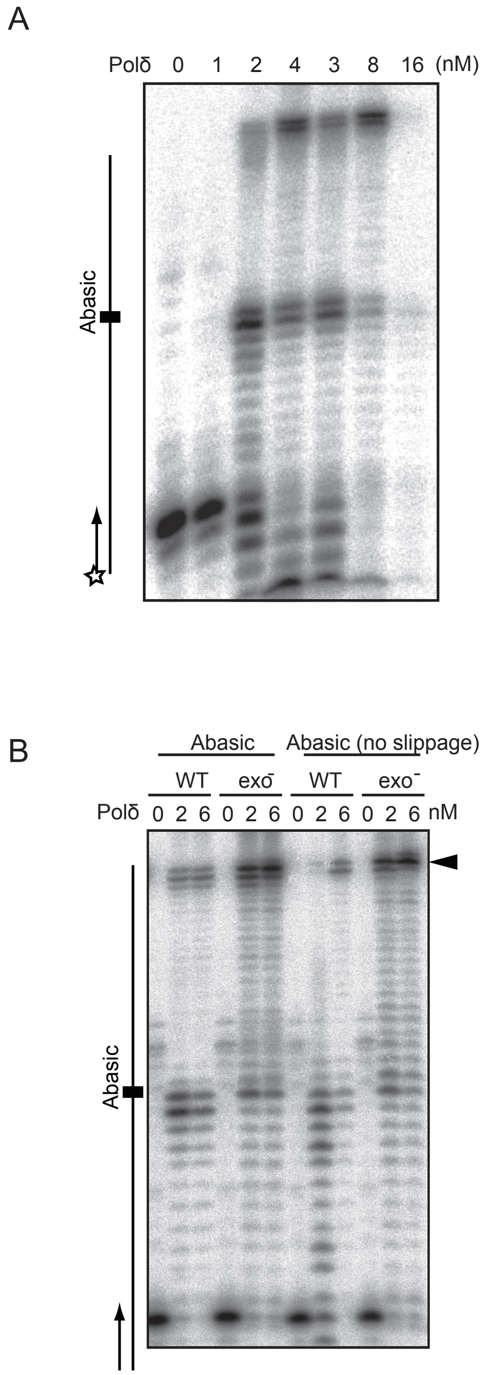
A
Polδ 0 1 2 4 3 8 16 (nM)
Abasic
B
Abasic
Abasic (no slippage)
WT exo⁻ WT exo⁻
Polδ 0 2 6 0 2 6 0 2 6 0 2 6 nM
Abasic

Supplementary Figure legends

Figure S1 Generation of cell lines expressing proofreading deficient POLD1

(A) Alignment of the proofreading exonuclease domain of Polδ by ClustalW (http://www.ebi.ac.uk/clustalw/). Amino acid residues are shaded according to BOXSHADE (http://www.ch.embnet.org/software/BOX-form.html). The asterisk represents the catalytic center of the exnonuclease domain of POLD1.

(B) Scheme for the knock in mutation of *POLD1* D402A in DT40 cells. The wild type chicken *POLD1* locus from intron 9 to exon 11 was indicated. A *hisD* selection-marker gene were inserted in intron 10. Targeting construct (bottom) are shown and compared with the relevant chicken *POLD1* genomic sequences (top). Solid boxes indicate the position of the exons. Relevant *PstI* sites and the position of the probe used in the Southern blot analysis are indicated. Black arrows indicate the position of primers used for RT-PCR in (E).

(C) Schematic representation of the generation of knock in mutation of *POLD1* D402A.

(D) Disruption of *POLD1* was confirmed by Southern blot.

(E) Insertion of the *POLD1* D402A mutation was confirmed by RT-PCR followed by restriction digestion by *Taq*I. D402A mutation is associated with disruption of *Taq*I recognition site.

Figure S2 Outline of the piggyBlock transposition-based system to analyze TLS past a CPD UV photoproduct

CPD placed opposite GpC mismatch in the piggyBlock plasmid carrying the puromycin

resistance (puro^R) gene. After transfection, we immediately did limiting dilution of the cells in 96-well cluster plates followed by the selection of clones carrying the piggyblock plasmid randomly integrated into the genome using puromycin. Bypass by accurate TLS inserts the correct complementary base (AA) on lower strand at the damaged template base. Alternatively, the nascent strand of the sister chromatid is used as an alternative undamaged template; one possible mechanism for such a template switching illustrated.

Figure S3 Ratio between TLS and Template switching events upon replication arrest at a CPD UV photoproduct

The pie charts indicate the percentage of TLS and template switching events.

Figure S4 Sequence of oligonucleotide primers and templates used in *in vitro* primer extension assay

(A) For abasic lesion bypass, we used 49 mer template containing abasic site (AP). 5' end of 17 mer primer was labeled with ^{32}P (asterisk).

(B) To test possible slippage event after insertion of A by Polδ opposite abasic site, we used 49 mer template containing abasic site (AP) where all T for template looping out was changed to C.

(C) For CPD lesion bypass, we used 30 mer template containing CPD (indicated by right triangle bellow two thymines). 5' end of 16 mer primer was labeled with ^{32}P (asterisk).

Figure S5 Bypass replication beyond abasic site by human Polδ holoenzyme

(A, B) DNA synthesis reactions were carried out with the proofreading proficient Polδ holoenzyme on abasic template and primer strands, which are schematically shown on the left. The 5’ end of the primers was ^{32}P labelled as shown by a star. We performed reactions in 5 µl reaction mixture containing 30 mM HEPES-NaOH (pH 7.4), 7 mM $MgCl_2$, 8 mM NaCl, 0.5 mM dithiothreitol, and 10 µM each dNTP in the presence of 15 nM of primer/template complex and 50 nM of PCNA for 15 min at 37 ºC.

(A) DNA synthesis reactions carried out with the indicated concentrations of wild type Polδ holoenzyme on template and primer strands.

(B) DNA synthesis reactions were carried out with 2 and 6 nM of the indicated holoenzymes. The sequences of the two template strands are shown in Figure S4.
